# Supplementary material for: Content-rich biological network constructed by mining PubMed abstracts
Source: BMC Bioinformatics. 2004 Oct 8;5:147. doi: 10.1186/1471-2105-5-147 (PMC528731; doi:10.1186/1471-2105-5-147)
Supplement: Additional File 5 — The original Chilibot query results of the term "long-term potentiation (LTP)" and 22 other terms, limiting the latest references analyzed to the years 1990, 1995, 2000, and 2004. [file 1471-2105-5-147-S5.bz2 › chilibotAdditionalFile5/ltp2000/html/PLC_ACTIN.html]

 


 **PLC** and **ACTIN** 
  
Found 79 abstracts in PubMed,  **30 abstracts were retrieved and analyzed**.  


---

 Search Google  |
 PDF files only 
|  EDU domain only 

---

**Interactive relationship** (e.g. stimulation, inhibition, etc)

- **PLC**  gamma1 signaling pathway and villin activation are involved in  **actin**  cytoskeleton reorganization induced by Na Pi cotransport up regulation.  Ref: 10949911 Mol Med, 2000
- Together, these data suggest that permanent sequential activation of both PI3K and PI  **PLC**  is necessary for the dramatic reorganization of the  **actin**  cytoskeleton in oncogene transformed fibroblasts, resulting in constitutive ruffling and macropinocytosis.  Ref: 11029048 Mol Biol Cell, 2000
- The formation of  **actin**  stress fiber by PMT seems to be stimulated through the indirect activation of Rho, which resides downstream of  **PLC** , PMT and DNT seem to elicit similar toxic effects, at least in part, through the activation of Rho.  Ref: 9560776 J Vet Med Sci, 1998
- In conclusion, our results demonstrate CCKA receptor activation causes rapid tyrosine phosphorylation of p130 through  **PLC**  dependent and independent mechanisms that require the participation of the small GTP binding protein Rho and the integrity of the  **actin**  cytoskeleton, but not the microtubule network.  Ref: 9931015 Biochemistry, 1999
- The loss of  **PLC**  activation was not the result of a decrease in EGF receptor or phospholipase C gamma1 PLCgamma1 protein levels, nor the result of a loss of tyrosine phosphorylation of these proteins, but was associated with a decrease in EGF induced translocation of PLCgamma1 to the Triton insoluble fraction, presumably reflecting binding to the  **actin**  cytoskeleton.  Ref: 11050044 Hepatology, 2000
- Since EGF receptor is also co localized with the cortical  **actin**  it is concluded that  **PLC**  gamma 1 co localized with  **actin**  is mediated by the EGF receptor.  Ref: 9610484 Tsitologiia, 1998
- After the treatment with cytochalasin B  **PLC**  gamma 1 is co localized with  **actin**  aggregates and cytoskeleton elements other than  **actin** .  Ref: 9610484 Tsitologiia, 1998
- **PLC**  gamma 1 is co localized only with the cortical  **actin**  but not with  **actin**  stress fibers.  Ref: 9610484 Tsitologiia, 1998
- The effectiveness of the  **actin**  based diffusion barrier is modulated by various signal pathways and effectors, most importantly, by the  **actin**  depolymerizing reorganizing activity of the phospholipase C  **PLC**  coupled Ca signaling.  Ref: 10362014 J Cell Physiol, 1999

**Parallel relationship** (e.g. studied together, co-existance, homology, etc.)

- The nascent models highlight the roles of various intracellular signaling pathways including phospholipase C gamma  **PLC**  gamma, phosphatidylinositol PI 3 kinase, mitogen activated protein MAP kinase, and  **actin**  cytoskeleton related events.  Ref: 10547668 Adv Cancer Res, 2000
- Although, tyrosine phosphorylation of  **PLC**  gammal was not observed during Na P i cotransport up regulation, genistein treatment abolished the enzyme s translocation to the  **actin**  cytoskeleton, as well as its association with villin.  Ref: 10949911 Mol Med, 2000
- Further analysis revealed that low concentrations of extracellular phosphate resulted in phospholipase Cgammal  **PLC**  gammal translocation to the  **actin**  cytoskeleton, without increases in its tyrosine phosphorylation.  Ref: 10949911 Mol Med, 2000
- When  **PLC**  gamma1 and F  **actin**  were double stained, the endogenous  **PLC**  gamma1 demonstrated a diffuse cytoplasmic staining and or perinuclear staining, while phalloidin staining demonstrated that all cells have filamentous cytoplasmic distribution of F  **actin** .  Ref: 10459043 Mol Vis, 1999
- The  **PLC**  inhibitor U73122 has no effect on this process, nor is the  **actin**  severing capacity of gelsolin required as EGF treatment reduces focal adhesions in gelsolin devoid fibroblasts, further supporting the contention that focal adhesion disassembly is signaled by a pathway distinct from that involving PLCgamma.  Ref: 9454735 J Cell Sci, 1998
- Tyrosine phosphorylation of PDGF Rbeta,  **PLC**  gamma1, ERK1 and ERK2, p125 FAK and paxillin as well as Sm alpha  **actin**  was examined by the chemiluminescence Western blotting method.  Ref: 10928958 Br J Pharmacol, 2000
